# Smoking and the Bier: Refusing to Operate if the Patient Keeps Smoking

**DOI:** 10.1016/j.atssr.2023.02.024

**Published:** 2023-03-16

**Authors:** Stephanie H. Chang, Mark B. Orringer, Robert M. Sade

**Affiliations:** 1 Division of Cardiothoracic Surgery, Department of Surgery, NYU Langone Medical Center, New York, New York; 2 Section of Thoracic Surgery, Department of Surgery, University of Michigan, Ann Arbor, Michigan; 3 Division of Cardiothoracic Surgery, Department of Surgery, Medical University of South Carolina, Charleston, South Carolina

## Introduction

Robert M. Sade, MD

Thoracic surgeons witness at close range the devastating effects of smoking tobacco. We also must deal with postoperative complications created or exacerbated by the damaging effects of current smoking on pulmonary and cardiovascular functioning. To mitigate such complications, we want patients to stop smoking before chest operations. But what should we do if the patient has tried to stop but has found that impossible?

Two surgeons with opposing views analyze and debate the following fictional vignette that focuses on the ethics of a patient’s inability to comply with steps needed to facilitate safe surgical procedures.

### The Case of the Smoker With Lung Cancer

Roland “Rolly” Marlborough is a 60-year-old man with chronic obstructive pulmonary disease (COPD), recently diagnosed with lung cancer. Imaging demonstrates a 2-cm nodule in the left lower lobe, and biopsy reveals squamous cell carcinoma; additional investigation showed it to be a cT1b N0 lesion. He smoked cigarettes at a rate of 2 packs a day for 30 years. He has tried various methods to stop smoking and was able to reduce his smoking to 1 pack a day, but he has been unable to stop entirely.

Thoracic surgeon Dr Orson Ringer explains to the patient that he must stop smoking—his survival risk will be significantly worse if smoking continues. Although the patient could more safely undergo stereotactic body radiation therapy (SBRT) rather than excision, his wife died during a course of radiation therapy and he refuses radiation, insisting on an operation.

Lengthy discussion of a surgical procedure’s higher risk and the patient’s aversion to radiation ends with the patient continuing to insist on an operation, even in the face of the increased risk related to his inability to stop smoking. Dr Ringer is not sure whether to proceed, so he asks 2 colleagues for their advice: should he operate on Mr Marlborough?

## PRO

Stephanie Chang, MD

Dr Ringer should operate on Mr Marlborough, even though he is unable to quit smoking, because surgical resection of a biopsy-proven lung cancer is still preferable to no medical intervention. Dr Ringer is asking what the correct thing is for this particular patient, who has failed prior attempts to quit smoking and refuses SBRT. With no other appropriate treatment options for a cT1b N0 lesion, not offering an operation is tantamount to refusing care for this patient. Our responsibility as physicians is to provide the best care for the patient, guided by the 4 pillars of ethics: autonomy, beneficence, nonmaleficence, and justice.

### Autonomy

Autonomy is defined as patients' having final decision-making about treatment while being fully informed of the risks and adverse effects of the treatment options. Dr Ringer has provided the patient with the pertinent risks and benefits, and he is aware that continued smoking will lead to increased risk of complications, including death, compared with if he quit smoking. He also knows that SBRT is safer in the short term, with minimal decrease in long-term survival. However, given his personal experience, Mr Marlborough is refusing to undergo SBRT. He has also tried many times to quit smoking, unsuccessfully.

Whereas it is our role to guide patients and to provide the information required, it is equally our role to protect the patient’s autonomy. Mr Marlborough is aware of the increased risk of surgical complications in him compared with a nonsmoker without COPD and, as the patient, is willing to bear this risk.

### Beneficence

Beneficence is doing what is best for the patient. Mr Marlborough has already refused SBRT. Dr Ringer has to decide whether operating will result in a better or worse outcome than not operating on biopsy-proven T1b N0 lung cancer.

Few studies have evaluated the natural history of untreated lung cancer. One study of the California Cancer Registry showed only a 9% 5-year survival in patients with biopsy-proven T1 lung cancer.^1^ Current 5-year survival for patients who present with clinical stage IA2 (T1b N0) is >80%.

Rodriguez and colleagues^2^ in 2017 showed that current smokers vs recent ex-smokers (quit within 16 weeks of a surgical procedure) have no significant difference in postoperative pulmonary complications after lobectomy, defined as postoperative atelectasis requiring bronchoscopy, pneumonia, or respiratory insufficiency (oxygen saturation <90% on room air). Patients' characteristics and cancer staging were matched, with complication rates of 4.5% (6/134) in smokers vs 3.7% (5/134) in recent ex-smokers (*P* = .76). There is likely to be an even lower risk of such complications for Mr Marlborough as he is a candidate for sublobar resection, given that his lesion is 2 cm.

### Nonmaleficence

Nonmaleficence is best known as “First, do no harm,” which includes harm that may come from neglect or nonaction. Although there is an increased risk of postoperative complications because of Mr Marlborough’s smoking and underlying COPD, the greater risk of overall harm is from nonaction. As stated previously, not operating carries significant risk, with only a 9% 5-year survival. Similarly, if Dr Ringer refuses to operate on Mr Marlborough, there is further harm by delaying care and damaging the physician-patient relationship.

The harm caused by delay in care is difficult to quantify. During the coronavirus 2019 (COVID-19) pandemic, patients were hesitant to seek normal medical care. A study in Spain demonstrated that during COVID-19, there were 38% fewer new lung cancer cases, whereas advanced stage disease increased from 46% to 58%, symptomatic disease increased from 63% to 74%, and >2 sites of metastatic disease at presentation increased from 12% to 16%.^3^ The consequence of this delay in care was decreased median overall survival and increased 30-day mortality (from 25% before COVID-19 to 49% during COVID-19).^3^ If Dr Ringer does not offer an operation and delays treatment for Mr Marlborough, progression of disease is likely.

Furthermore, the physician-patient relationship will be damaged if Dr Ringer refuses to operate on Mr Marlborough because of his smoking status. Many smokers believe that physicians treat smokers differently with decreased respect and so conceal their smoking status from physicians. If Dr Ringer does not offer the operation, the patient will have increased distrust of the medical profession. This distrust can lead to further delay in presentation or increased harm if he conceals his smoking status from other physicians.

### Justice

Justice involves equal distribution of resources with the same quality of care for all patients. If Dr Ringer does not offer current smokers the option for surgical resection, he is discriminating against a large subset of the population. In the United States, 22% are former smokers, 21% are current smokers, and 20% are sporadic or occasional smokers.^4^ Moreover, not operating on patients who smoke targets patients with a medical disorder. Smoking is considered to be an addiction. Mr Marlborough has tobacco dependence, as shown by his prior inability to stop smoking despite actively trying. In addition, denying care to smokers affects a vulnerable population. Tobacco dependence is higher among veterans, people with low income, people with low levels of education, and patients with other psychiatric disorders. Not offering the same level of medical care to smokers is neither ethical nor just.

In conclusion, Dr Ringer should operate on Mr Marlborough. The patient has medical decision-making capacity and refuses SBRT. Dr Ringer should respect Mr Marlborough’s decision to not undergo SBRT. In that situation, sublobar surgical resection of the biopsy-proven cT1b N0 lung cancer will result in a better outcome compared with no treatment.

## CON

Mark Orringer, MD

Dr Ringer should not operate on Mr Marlborough because he is still smoking. It takes only a few weeks of careful preoperative assessment and conditioning for the patient to reduce postoperative morbidity and mortality with no negative oncologic effect. My unwritten contract with new patients emphasizes the team effort needed for a successful outcome. I tell patients, “I’m half the team, and you are the other half.” What *they* have to do to get through what lies ahead is complete abstinence from cigarette smoking for at least 2 to 4 weeks, daily use of an incentive inspirometer, and walking 2 to 3 miles a day. Without their part of the team effort, I will not be their surgeon and am not willing to accept their added risk and complications that might occur. Patients and families understand this discussion, and compliance is the rule, not the exception.

With his COPD, Mr Marlborough is a high-risk patient. Many COPD patients with chronic airway irritation from smoking have increased cough and sputum production that may worsen for 1 to 2 weeks after an operative procedure—“reflex bronchorrhea”— contributing to atelectasis and pneumonia. Most publications that report the impact of smoking and smoking cessation on the outcomes of pulmonary resections document a statistically significant increased incidence of postoperative pulmonary and cardiac complications and hospital mortality in active smokers undergoing pulmonary resection.^5^^,^^6^ These studies strongly reinforce the recent ERAS (Enhanced Recovery After Surgery) protocol reviews, which place smoking cessation before thoracic surgery among the strongest recommendations for our specialty.^7^

### Ethical Considerations

It may be argued that Mr Marlborough’s cigarette smoking is a tobacco use disorder, an addiction that he cannot just stop, so one cannot ethically deny an operation. I do not agree. In my many years of operating on such patients, I have told the very recalcitrant that I was not asking them to give up smoking forever, but to get them through their operation safely; whereas it might not be pleasant if withdrawal symptoms were to occur, they needed to stop for several weeks. And this clarification has generally been successful.

How does this case relate to the fundamental principles of medical ethics? *Beneficence* is the obligation of the physician to act for the benefit of the patient. Performing a pulmonary resection in Mr Marlborough despite the increased operative risk posed by his severe COPD and active smoking without mitigating the latter is *not* in his best interest. Because of his marginal pulmonary function, some might be tempted to perform a minimally invasive wedge resection of this early-stage lung cancer. The surgical approach, however, does not mitigate the increased risk as video-assisted thoracic surgical lobectomy still carries a risk of postoperative pulmonary complications, and only current smoking is a significant independent risk factor.^8^

The principle of *nonmaleficence* is the obligation not to harm the patient. If Dr Ringer chooses to proceed with a pulmonary resection in this active smoker, he will be potentially contributing to postoperative atelectasis and pneumonia.

What about the principle of *autonomy*—Mr Marlborough’s right to choose to have an operation or not? Dr Ringer has explained to him that his COPD and smoking increase his perioperative risk. Mr Marlborough has been informed that there is an effective nonoperative option (SBRT). Is he still entitled to insist on surgical therapy? The right of the patient to self-determination, as with all ethical principles, must be balanced against competing moral principles. Dr Ringer would be violating his commitment to beneficence—acting for the benefit of the patient—if he agrees to operate despite his better judgment. He should refuse to operate unless Mr Marlborough accepts the mandate to stop cigarette smoking for at least 2 to 4 weeks. If he is not agreeable to this course, Dr Ringer should express his willingness to refer Mr Marlborough to a thoracic surgery colleague for a second opinion and management.

### Closing Comments

The contribution of careful preoperative assessment and preparation of our patients for a successful surgical outcome cannot be overestimated. Mr Marlborough’s COPD and marginal pulmonary function place him at increased risk for postoperative complications after any type of pulmonary resection. He should be required to commit to being a partner in the surgical team by complete abstinence from cigarette smoking for at least 2 to 4 weeks, daily use of an incentive inspirometer, and walking 2 to 3 miles a day. *We* are the captains of the ship. *We*, not the patient, are ultimately responsible for complications or death that follows inadequate preoperative preparation. No surgeon should be bullied by a patient, family, or other physician to undertake an operation he or she does not believe is as safe for that patient as it could be and should be.

I believe that it is not only ethical to refuse to do elective lung operations on an active cigarette smoker but also unethical to *do* an elective pulmonary resection in such a patient: accepting increased risk without mitigating it, cutting corners, potentially harming the patient, and violating the ethical imperatives to act for the benefit of the patient and to do no harm. There really should be no debate about this one!

## Concluding Remarks

Robert M. Sade, MD

The essayists in this debate reach starkly different conclusions about what Dr Ringer should do, although they agree on several points. Both Chang and Orringer use the ethical approach of Beauchamp and Childress’s 4 principles^9^ for their analysis, although they interpret and apply those principles differently. Both accept that an operative procedure poses a higher risk of complications than focused radiation treatment for Mr Marlborough’s lung cancer. Both act in what they believe to be the patient’s best interest, but they diverge in seeing where that interest lies.

During the last few decades, the idea of shared decision-making has gained support in both the health care and the lay communities. In that model, medical decisions are made through collaboration between the physician, who supplies factual information about the patient’s medical condition and offers advice about reasonable alternative treatments, and the patient, who evaluates the facts in the context of his or her own value system.^10^ Both essayists recognize that Dr Ringer has engaged in dialogue with the patient, and both support that engagement. Chang sees that process as an instance of shared decision-making and recommends accepting the patient’s decision. Orringer, to the contrary, believes that his professional commitment to patient safety weighs more heavily than the patient’s autonomous decision and so recommends refusing to accept the patient’s choice.

Is Chang being too accommodating to the patient? Is Orringer being too harsh in rejecting the patient’s decision? Readers must decide for themselves.
